# Antioxidant agents for delaying diabetic kidney disease progression: A systematic review and meta-analysis

**DOI:** 10.1371/journal.pone.0178699

**Published:** 2017-06-01

**Authors:** Davide Bolignano, Valeria Cernaro, Guido Gembillo, Rossella Baggetta, Michele Buemi, Graziella D’Arrigo

**Affiliations:** 1 CNR- Institute of Clinical Physiology, Reggio Calabria, Italy; 2 Chair of Nephrology, Department of Clinical and Experimental Medicine, University of Messina, Messina, Italy; Universita degli Studi di Perugia, ITALY

## Abstract

**Background:**

Oxidative stress is a key player in the genesis and worsening of diabetic kidney disease (DKD). We aimed at collecting all available information on possible benefits of chronic antioxidant supplementations on DKD progression.

**Study design:**

Systematic review and meta-analysis.

**Population:**

Adults with DKD (either secondary to type 1 or 2 diabetes mellitus)

**Search strategy and sources:**

Cochrane CENTRAL, Ovid-MEDLINE and PubMed were searched for randomized controlled trials (RCTs) or quasi-RCTs without language or follow-up restriction.

**Intervention:**

Any antioxidant supplementation (including but not limited to vitamin A, vitamin C, vitamin E, selenium, zinc, methionine or ubiquinone) alone or in combination.

**Outcomes:**

Primary outcome was progression to end-stage kidney disease (ESKD). Secondary outcomes were change in albuminuria, proteinuria, serum creatinine and renal function.

**Results:**

From 13519 potentially relevant citations retrieved, 15 articles referring to 14 full studies (4345 participants) met the inclusion criteria. Antioxidant treatment significantly decreased albuminuria as compared to control (8 studies, 327 participants; SMD: -0.47; 95% CI -0.78, -0.16) but had apparently no tangible effects on renal function (GFR) (3 studies, 85 participants; MD -0.12 ml/min/1.73m^2^; 95% CI -0.06, 0.01). Evidence of benefits on the other outcomes of interest was inconclusive or lacking.

**Limitations:**

Small sample size and limited number of studies. Scarce information available on hard endpoints (ESKD). High heterogeneity among studies with respect to DKD severity, type and duration of antioxidant therapy.

**Conclusions:**

In DKD patients, antioxidants may improve early renal damage. Future studies targeting hard endpoints and with longer follow-up and larger sample size are needed to confirm the usefulness of these agents for retarding DKD progression.

## Introduction

Diabetes Mellitus (DM) remains one of the most challenging global epidemics of the twenty-first century. More than 350 million people worldwide are estimated to be affected by this metabolic disorder [1]. Diabetic kidney disease (DKD) currently ranks as the first cause of end-stage kidney disease (ESKD), accounting for approximately 50% of cases in the developed world [2]. As many as 50% of individuals with longtime DM usually develop some degree of renal damage during their lifetime [3]. Progressive impairment in renal function is associated with an increased risk of cardiovascular events and hospitalizations, particularly in ESKD patients needing chronic renal replacement therapy by dialysis or kidney transplantation. Current strategies available for slowing-down DKD progression largely failed to achieve stable results in the long term. Alternative or additive approaches for maximizing reno-protection are thus eagerly advocated [4]. It is nowadays well recognized that oxidative stress plays a major role in the genesis and worsening of DKD [5]. A persistent state of hyperglycemia and the increase in advanced glycation end products (AGEs) elicit the generation of reactive oxygen species (ROS) which, in turn, enhance chronic inflammation and glomerular and tubular hypertrophy, eventually impairing overall renal function. Sparse evidence has now accrued indicating that antioxidant supplements may bring significant benefits to DKD patients, including the reduction of urinary albumin and total protein excretion and the normalization of glomerular filtration rate [6]. This raises the question as to whether such supplements should be systematically recommended for improving reno-protection in diabetic patients, particularly with early signs of renal damage.

We therefore aimed at performing a systematic review and meta-analysis of randomized clinical trials to investigate whether chronic antioxidant supplementations may represent a potential tool for slowing down disease progression in patients with diabetic kidney disease.

## Methods

This review follows PRISMA guidelines [7] for reporting in systematic reviews and meta-analysis and has been performed according to a previously published protocol [8]

### Data source and search strategy

Ovid-MEDLINE, PubMed and CENTRAL databases were searched for articles without time or language restriction up to November 15, 2016 using high sensitive search strategies (S1 Table). References from pertinent studies and eminent full-reviews were screened for additional articles. The search was designed and performed by three Authors (DB, GB and RB).

### Study selection and data extraction

We included any randomized controlled trial (RCT) and quasi-RCT (trials in which allocation to treatment was made by alternation, use of alternate medical records, date of birth or other expected methods) testing the effects of any antioxidant supplementation (including but not limited to vitamin A, vitamin C, vitamin E, selenium, zinc, methionine or ubiquinone, either alone or in combination to other treatments) on renal endpoints in patients with diabetic kidney disease (DKD).

Studies were considered regardless of dosage or duration of administration of antioxidants and type of comparator. For cross-over studies the first period was considered.

DKD was defined as evidence of renal damage (chronic kidney disease, CKD) related to diabetic disease. CKD, in turn, was defined according to the National Kidney Foundation-Kidney Disease Outcomes Quality Initiative (NKF KDOQI) guidelines by the presence of a reduced glomerular filtration rate (GFR) <90 mL/min/1.73 m^2^ or the persistence of hyperfiltration and/or urinary abnormalities such as pathological albuminuria, proteinuria or hematuria in subjects with GFR≥90 mL/min/1.73 m^2^ [9].

The primary endpoint of interest was progression to end-stage kidney disease (ESKD), defined as need for chronic renal replacement therapy, kidney transplantation or doubling of serum creatinine from baseline values. Secondary outcomes were change in albuminuria, proteinuria, serum creatinine and renal function (creatinine clearance/eGFR). Information on any adverse event was also collected, when reported.

Studies were excluded if: 1) dealing with diabetic patients with no evidence of kidney disease or dealing with CKD patients without diabetes; 2) focusing on DKD patients on chronic or acute renal replacement therapy (e.g. hemodialysis or peritoneal dialysis); 3) testing the effects of synthetic antioxidants (e.g. bardoxolone methyl) or antioxidant mixtures which exact composition was not defined; 4) not providing short or long-term data on the outcomes of interest.

Studies where at least part of the population satisfied the above criteria were included in the review.

Titles and abstracts were screened independently by two authors (VC, GG) who discarded studies that were not pertinent to the topic. Case reports, reviews, editorials and studies performed on children (age<18) were excluded from qualitative analyses but screened for potential additional references. Two Authors (VC, GG) independently assessed the retrieved abstracts and the full text of these studies to determine eligibility according to the inclusion/exclusion criteria.

A third reviewer (GD) solved possible discrepancies on study judgments. Data extraction and analysis were carried out by two reviewers (VC, GG) and independently verified by another (GD).

### Data analysis

Pooled meta-analyses were performed for outcomes in which data were available from more than two studies. Data on outcomes reported by single studies or in a descriptive way were reported narratively. The effects of treatment on continuous variables were assessed as mean difference (MD) or standardized mean difference (SMD), as appropriate.

Data were pooled using the random-effects model. To ensure robustness of the model and susceptibility to outliers pooled data were also analyzed with the fixed-effects model. Data expressed as median and range were converted to mean and SD by applying the Hozo formula [10]. Heterogeneity was assessed by the Chi^2^ test on N-1 degrees of freedom, with an alpha of 0.05 considered for statistical significance and the Cochrane-I^2^ [11]. I^2^ values of 25%, 50% and 75% were considered to correspond to low, medium and high levels of heterogeneity, respectively. Possible sources of heterogeneity were explored by sensitivity analysis.

Publication bias was evaluated by the Egger’s regression test and by visual inspection of funnel plot. Statistical analyses were performed by GD, VC and GG using Review Manager (RevMan; Version 5.3. Copenhagen: The Nordic Cochrane Centre, The Cochrane Collaboration, 2014) and Stata/IC (Version 13.1, Stata Corp LP, Texas, USA).

### Risk of bias assessment

Likelihood of bias in the single RCTs was evaluated according to the checklist developed by the Cochrane Renal Group that considers the presence of potential selection bias (random sequence generation and allocation concealment), performance bias (blinding of investigators and participants), detection bias (blinding of outcome assessors), attrition bias (incomplete outcome data), reporting bias (selective reporting) and possible other sources of bias.

### Summary of findings and quality of the evidence

A “Summary of findings” table summarizing pooled evidence for the main outcomes was constructed according to the GRADE method [12]. The five GRADE considerations (study limitations, consistency of effect, imprecision, indirectness and publication bias) were taken into account to assess the quality of a body of evidence for the main pre-specified outcomes. All decisions to downgrade or upgrade the quality of studies were justified using footnotes and comments were made, when appropriate, to help readers' understanding of the review.

## Results

### Search results

**Fig 1:**
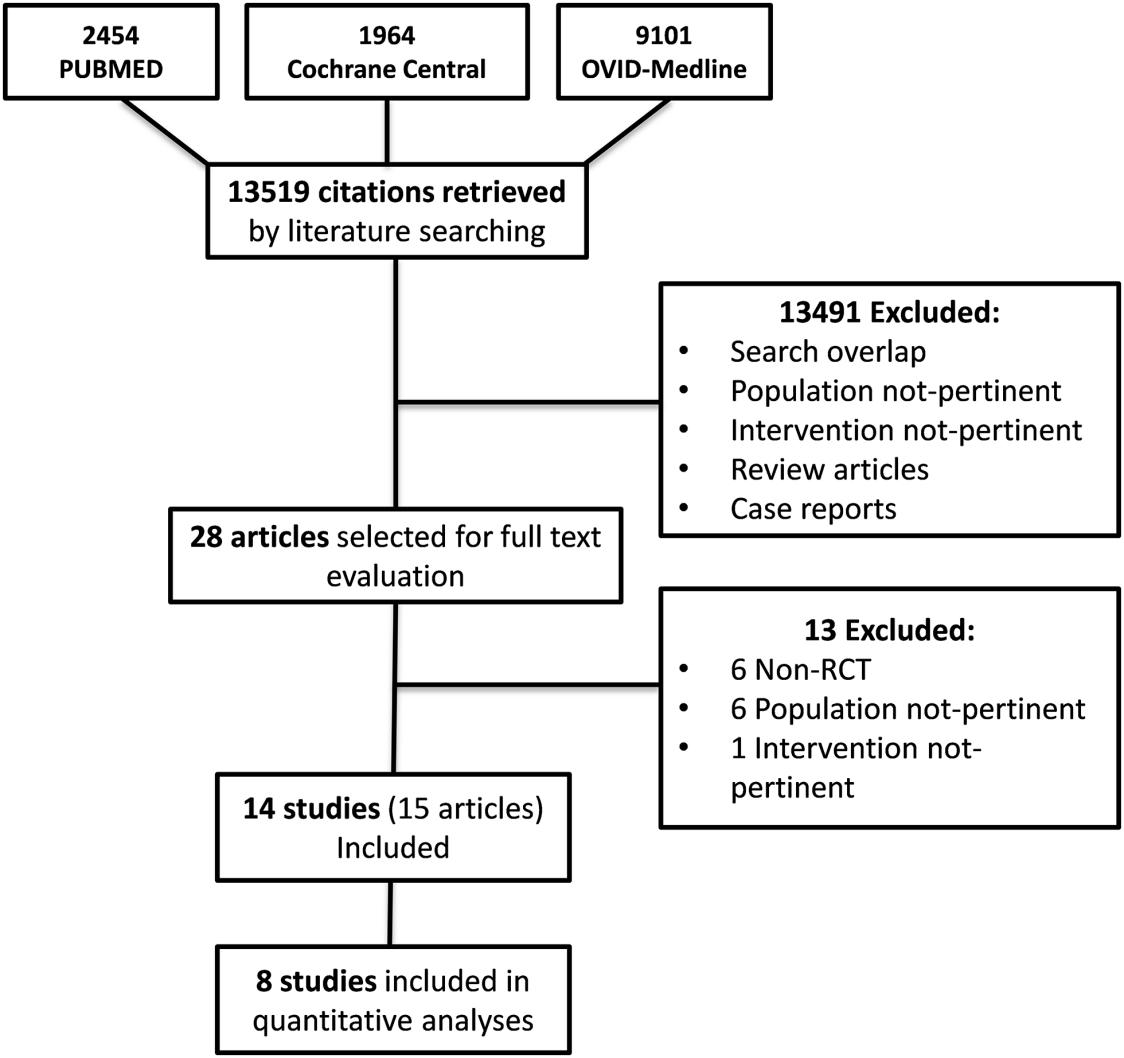
Study selection flow.

Fig 1 shows the flow diagram of the study selection process. Thirteen thousand five hundred and nineteen potentially relevant citations were initially found. By screening titles and abstracts, a total of 13491 references were excluded for various reasons (search overlap, study population or intervention not pertinent, review articles or case reports). Amongst the 28 studies selected for full text examination, 13 studies were excluded because not RCTs (n = 6) or because the study population (n = 6) or the intervention (n = 1) did not fulfil the review criteria. A total of 15 articles referring to14 full studies (4345 participants) were reviewed in detail. Eight studies [13–20] (327 participants) providing suitable numerical data on the outcomes of interest contributed to pooled meta-analyses. Main characteristics of the included studies are described in Table 1.

**Table 1:** Summary of main characteristics and findings of the included studies.

Study, year (ref)	Study population	Population characteristics	Intervention	Control	Vintage	Outcome(s)	Results	Notes
Klein et al. 1995 [13]	Normoalbuminuric IDDM patients with hyperfiltration	N = 24 - Male = 100% - Age = ~ 32.5 yrs - Diabetes vintage = ~7.5 yrs - GFR~141.5 ml min^-1^ 1.73m^2^	Vitamin C (3 g twice a day) (n = 12)	Placebo (n = 11)	4 wks	UAE (μg min^-1^)	-No significant difference between intervention and control group (7.4±2.1 vs 7.4±2.8)	- Double-blind - One drop-out from placebo group for stomachache - No adverse events in the intervention group reported
						GFR (ml min^-1^ 1.73m^2^)	-No significant difference between intervention and control group (134±12 vs 137±16)	
Sinclair et al 1997 [21]	NIDDM patients with microalbuminuria	N = 14 - Male = 57% - Age = 65 yrs	Vitamin C (750 mg/day) Vitamin E (600 mg/day) Beta-carotene (18 mg/day) (n = 6)	Placebo (n = 4)	12 wks	UAE (μg min^-1^)	- Average change over active period (-201±0.23) and over placebo period (0.054±0.15) (p = 0.0054)	- Cross-over - Four drop-out - Six patients received treatment for 6 wks in each arm
McAuliffe et al 1998 [22]	IDDM and NIDDM patients with microalbuminuria	N = 20 -Male = 75% -Age = ~58 yrs - Diabetes vintage = ~12 yrs - NIDDM = 90% -UAE = ~60 μg min	Vitamin C (500 mg twice a day) (n = 10)	Placebo (n = 10)	12 mo	UAE (μg min^-1^)	-Significant decrease in intervention group (p = 0.03)	- Double-blind -One drop-out from intervention group for bladder carcinoma
Gaede et al 2001 [14]	NIDDM patients with microalbuminuria	N = 29 -Male = 69% -Age = 58.7±7.3 -Diabetes vintage = 12.2±4.4 yrs	Vitamin C (1250 mg/day) Vitamin E (680 IU) (n = 29)	Placebo (n = 29)	4 wks	UAE (mg/24 h)	-Significant decrease in intervention group (19%, 95% CI 6–34%; p = 0.04)	-Double-blind -Cross-over -No adverse events reported
						Serum Cr (μmol/L)	-No difference between intervention (85±20) and placebo group (86±19; p = 0.55)	
Yokoyama et al 2001 [23]	NIDDM patients with microalbuminuria	N = 54 Not specified	Vitamin E (600 mg/day) (n = 29)	No treatment (n = 25)	6 mo	UAE (mg/day)	- No significant difference between intervention and placebo group	-Open label
Lonn et al 2002 [24]	IDDM and NIDDM patients	N = 3654 - Male = 63% - Age = ~65.4 - Diabetes vintage = ~11.45 yrs - NIDDM = ~97.75% - 31.6% with microalbuminuria	Vitamin E (400 IU/day) (n = 1838)	Placebo (n = 1816)	Average 4.5 yrs	Dialysis (%)	- No significant difference between intervention (0.5) and placebo group (0.5) (p = 0.97)	- Double-blind - No adverse events reported
						UAE (mg/g Cr)	- No significant difference between groups	
						Incidence of new-onset microalbuminuria (%)	- No significant difference between intervention (35.3) and placebo group (37.5) (p = 0.14)	
Farvid et al 2005 [15]	NIDDM patients with microalbuminuria	N = 76 - Male = 47% - Age = 50.5 yrs - Diabetes vintage = ~8.5 yrs - UAE = ~31.5 mg/g cr	-Group M (Zinc sulphate 15 mg + magnesium oxide 100 mg (n = 18) -Group V (Vitamin C 100 mg + Vitamin E 50 IU) (n = 20) -Group MV (Zinc sulphate 15 mg + magnesium oxide 100 mg + Vitamin C 100 mg + Vitamin E 50 IU) (n = 19)	Placebo (n = 19)	3 mo	UAE (mg/g Cr)	- Significant decrease in group MV (29.3 vs 10.8, p = 0.005) - Significant decrease in group V (35.6 vs 22.1, p = 0.034)	- Double-blind - Two drop-out for side effects - Five excluded from statistical analysis because of protocol violation
						Urinary protein (g/g cr)	- No within-group significant difference	
Giannini et al 2007 [16]	IDDM patients with microalbuminuria	N = 10 - Male = 70% - Age = 18.9±2.9 yrs - Diabetes vintage = 12.6±3.4 yrs - UAE = 33.4±7.6	Vitamin E 1200 mg/day (n = 10)	Placebo (n = 10)	6 mo	UAE (μg min^-1^)	- No significant difference between intervention group (24.35±8.67) and placebo group (27.29±11.89) (p = 0.59)	- Double-blind - Cross-over - No adverse events reported
						CrCl (μg min^-1^/1.73 m^2^)	- No significant difference between intervention group (154.6±29.1) and placebo group (155.6±29.3) (p = 0.34)	
Parham et al 2008 [17]	NIDDM patients with microalbuminuria	N = 50 - Male = 57% - Age = ~53 yrs - Diabetes vintage = ~11.2 yrs - UAE = ~88.5 mg/g cr	Zinc (30 mg/day) (n = 21)	Placebo (n = 21)	3 mo	UAE (mg/g Cr)	- Significant decrease in intervention group (86±57 vs 75±71) (p = 0.05) - No significant difference in placebo group - Significant difference between intervention (75±71) and placebo group (90±60) (p<0.05)	- Double-blind - Cross-over - Eight drop-out: Intervention group: four (one for epigastric pain, one for change of drugs, two for poor compliance) Control group: four (one for epigastric pain, one for change of drugs, two for starting insulin therapy)
						GFR (ml/min)	- No significant difference within intervention (88±29) and placebo group (83±20)	
Abarghouei et al 2012 [25]	NIDDM patients with macroalbuminuria	N = 60 Not specified	Sylimarin (420 mg/day) (n = 30)	Placebo (n = 30)	3 mo	UAE (mg/day)	- Significant within-group decrease - Higher decrease in the intervention vs control group (p = 0.005)	- Double-blind
Khan et al 2013 [18]	NIDDM patients with microalbuminuria	N = 54 - Age = ~56 yrs - Diabetes vintage = ~9 yrs - UAE = ~146 mg/day	Zinc (50 mg/day) (n = 23)	No treatment (n = 21)	12 wks	UAE (mg/day)	- Significant decrease in intervention group (146.87±30.83 vs 80.70±33.99) (p<0.0001) - Significant increase in control group (145.05±45.97 vs 157.43±49.51) (p = 0.02)	- 10 drop-out: 4 Intervention group: four (change of drug) 6 Control group: six (poor compliance)
Noori et al 2013 [19]	NIDDM patients with microalbuminuria	N = 34 - Male = 38% - Age = ~60.5 yrs - Diabetes vintage = ~13.5 yrs - UAE = ~208.5 mg/g cr	Lipoic acid (800 mg) + pyridoxine (80 mg)/day (n = 17)	Placebo (n = 17)	12 wks	UAE (mg/g cr)	- Significant decrease in intervention group (236±75 vs 162±44) (p<0.05) - Significant difference between groups (p<0.05)	- Double-blind, - No adverse events reported
Haghighat et al 2014 [20]	NIDDM patients with microalbuminuria	N = 50 - Male = 27% - Age = ~ 55 yrs - Diabetes vintage = ~4.7 yrs - UAE = ~ 20 nmol/dl	Tocotrienol-enriched canola oil (15 ml/day) (n = 23)	Placebo (n = 22)	4 wks	UAE (nmol/dl)	- Significant difference between groups (intervention: Median 11 IR: 9–25, control: Median 22 IR: 15–39.75; p<0.001)	- Double-blind -Five drop-out: Intervention group: two (one for lost to follow-up, one for changing in treatment protocol); Control group: three (one for inability to walk to centre, one for unavailability of 24h urine collection, one for unwillingness) - No adverse events reported
Jadhav et al 2014 [26]	Patients with diabetic nephropathy	N = 216 Not specified	- Vitamin C + Vitamin E - Reduced glutathione - Vitamin C + Vitamin E + Reduced glutathione	No treatment	4 mo	UAE (mg/g cr)	- Significant decrease in vitamins C + E group (33.2±2 vs 30.5±2; p<0.009) - Significant decrease in reduced glutathione group (31.4±2 vs 30.1±1; p<0.002) - Significant decrease in the vitamins plus glutathione group (33.3±2 vs 27.2±2; p<0.001)	- Open label - Sample size and dose of treatments in each group not specified

Cr: creatinine; CrCl: creatinine clearance; GFR: glomerular filtration rate; IDDM: insulin-dependent diabetes mellitus; IR: interquartile range; MO: months; NIDDM: non-insulin-dependent diabetes mellitus; UAE: urinary albumin excretion rate; wks: weeks; yrs: years;

### Study characteristics

Among the 14 RCTs reviewed [13–27], four had a crossover design [14, 16, 17, 21]. One study was multicenter [24, 27]. The study population ranged from ten [16] to 3654 [24] participants.

Ten studies [13–17, 19, 20, 22, 24, 25] were double-blind and three were open label [18, 23, 26]; in another study [21] blinding of participants and personnel was not specified.

Two RCTs [13, 16] enrolled subjects with insulin-dependent diabetes mellitus (IDDM), nine studies [14, 15, 17–21, 23, 25] were conducted on individuals with non-insulin-dependent diabetes mellitus (NIDDM) and two studies [22, 24] included both IDDM and NIDDM patients. In Jadhav et al [26] information on the type of diabetes was not provided. Study follow-up varied from 4 weeks [13, 14, 20] to 4.5 years [24]. The mean age of patients ranged from 18.9 [16] to 65.4 [24] years. Male gender spanned from 27% [20] to 100% [13]. Diabetes vintage varied from 4.7 [20] to 13.5 [19] years. Single antioxidant therapy was vitamin C [13, 22], vitamin E [16, 20, 23, 24], zinc [17, 18] and sylimarin [25]. In two studies [14, 26] patients received vitamin C in combination with vitamin E. One study [19] tested the effects of lipoic acid in combination with pyridoxine while in another one [21] vitamin C was combined to vitamin E and beta-carotene. Farvid et al. [15] tested the effects of a combined regimen of zinc and magnesium against vitamin C, vitamin E and the combination of both substances. Eleven studies [13–17, 19–22, 24, 25] compared antioxidants to placebo while in three studies [18, 23, 26] the control group did not take any treatment.

The daily dose of administered vitamin C ranged from 500 mg [22] to 6 g [13]; the dose of vitamin E varied from 400 [24] to 1200 mg [16]; zinc supplements ranged from 30 [15, 17] to 50 mg [18] lipoic acid was administered at a daily dose of 800 mg [19]. Jadhav et al. [26] did not provide information on the administered dose of antioxidants.

### Risk of bias

Risk of bias of RCTs is summarized in Table 2. Information of the random sequence generation was provided in five studies [15, 17, 19, 20, 24], as well as allocation concealment [13, 15–17, 22].

**Table 2:** Risk of bias in included studies.

Study, year (ref)	Random sequence generation	Allocation concealment	Blinding of participants and personnel	Blinding of outcome assessors	Incomplete outcome data	Selective reporting	Other sources of bias
Klein et al. 1995 [13]	**Unclear** (not stated)	**Low risk** (“the tablets were identical in size and color. The taste was blinded by the enterosoluble cover”)	**Low Risk** (double blind)	**Unclear** (not stated)	**Low risk** (One drop-out)	**Unclear**	**None known**
Sinclair et al 1997 [21]	**Unclear** (not stated)	**Unclear** (not stated)	**Unclear** (not stated)	**Unclear** (not stated)	**High risk** (29% drop-out)	**Unclear**	**None known**
McAuliffe et al 1998 [22]	**Unclear** (not stated)	**Low risk** (“identical placebo tablets”)	**Low Risk** (double blind)	**Unclear** (not stated)	**Low risk** (One drop-out)	**Unclear**	**None known**
Gaede et al 2001 [14]	**Unclear** (not stated)	**Unclear** (not stated)	**Low Risk** (double blind)	**Unclear** (not stated)	**Low risk** (no drop-out)	**Unclear**	**None known**
Yokoyama et al 2001 [23]	**Unclear** (not stated)	**Unclear** (not stated)	**High Risk** (open label)	**High Risk** (open label)	**Low risk** (no drop-out)	**Unclear**	**None known**
Lonn et al 2002 [24]	**Low risk** (central telephone randomization)	**Unclear** (not stated)	**Low Risk** (double blind)	**Unclear** (not stated)	**Low risk** (no drop-out)	**Low risk** (all the specified outcomes have been reported)	**High risk** (Industry-Funded)
Farvid et al 2005 [15]	**Low risk** (“block randomization procedure”)	**Low risk** (“the supplement and placebo capsules looked identical”)	**Low Risk** (double blind)	**Unclear** (not stated)	**Low risk** (two drop-out, five excluded from statistical analysis)	**Unclear**	**None known**
Giannini et al 2007[16]	**Unclear** (not stated)	**Low risk** (“Vitamin E and placebo were capsules of the same size, shape and color”)	**Low Risk** (double blind)	**Unclear** (not stated)	**Low risk** (no drop-out)	**Unclear**	**None known**
Parham et al 2008[17]	**Low risk** (“card-shuffling” randomization)	**Low risk** (“The placebo capsules were made to appear the same as the zinc capsules, in size, shape and color”)	**Low Risk** (double blind)	**Unclear** (not stated)	**Low risk** (16% drop-out equally distributed in both groups)	**Unclear**	**None known**
Abarghouei et al 2012 [25]	**Unclear** (not stated)	**Unclear** (not stated)	**Low Risk** (double blind)	**Unclear** (not stated)	**Low risk** (no drop-out)	**Unclear**	**None known**
Khan et al 2013[18]	**Unclear** (not stated)	**Unclear** (not stated)	**High risk** (open label)	**Unclear** (not stated)	**Low risk** (18.5% drop-out: 22% in the control group; 15% in the intervention group)	**Unclear**	**None known**
Noori et al 2013[19]	**Low risk** (“Patients…were randomly allocated … by blocked randomization”)	**Unclear** (not stated)	**Low Risk** (double blind)	**Unclear** (not stated)	**Low risk** (no drop-out)	**Unclear**	**None known**
Haghighat et al 2014 [20]	**Low risk** (“participants were assigned into two groups randomly by using a random number table”)	**Unclear** (not stated)	**Low Risk** (double blind)	**Unclear** (not stated)	**Low risk** (10% drop-out: 8% in the intervention group, 12% in the control group)	**Low risk** (all the specified outcomes have been reported)	**None known**
Jadhav et al 2014[26]	**Unclear** (not stated)	**Unclear** (not stated)	**High Risk** (open label)	**High Risk** (open label)	**Unclear** (not stated)	**Unclear**	**None known**

Ten RCTs were double blind [13–17, 19, 20, 22, 24, 25] and two studies were open label [23, 26]. In two studies, blinding was unclear [18, 21]. Attrition bias was low in all but one study [21] in which the overall drop-out rate was as high as 29%. Reporting bias was low in two studies [20, 24] and unclear in the remainder. Risk of funding bias was potentially high in one study [24]. No further sources of bias were identified.

### Outcome data

Data on progression to end-stage kidney disease defined as chronic renal replacement therapy was available in only one RCT [24]. Conversely, information on the need for kidney transplantation or doubling of serum creatinine was not available in any of the included trials. Change in urinary albumin excretion was analyzed in all RCTs [13–26]. Two studies, respectively, provided data on change in proteinuria [14] and serum creatinine [15]. Three studies [13, 16, 17] reported information on renal function. Data on adverse events were available in eight RCTs [13–17, 19, 20, 24].

### Effects of antioxidant supplements on primary outcomes

In Lonn [24] et al., the number of patients experiencing needing to start chronic dialysis due to DKD progression was not different between the intervention and the control group (p = 0.96).

### Effects of antioxidant supplements on secondary outcomes

#### Urinary albumin excretion (UAE)

**Fig 2:**
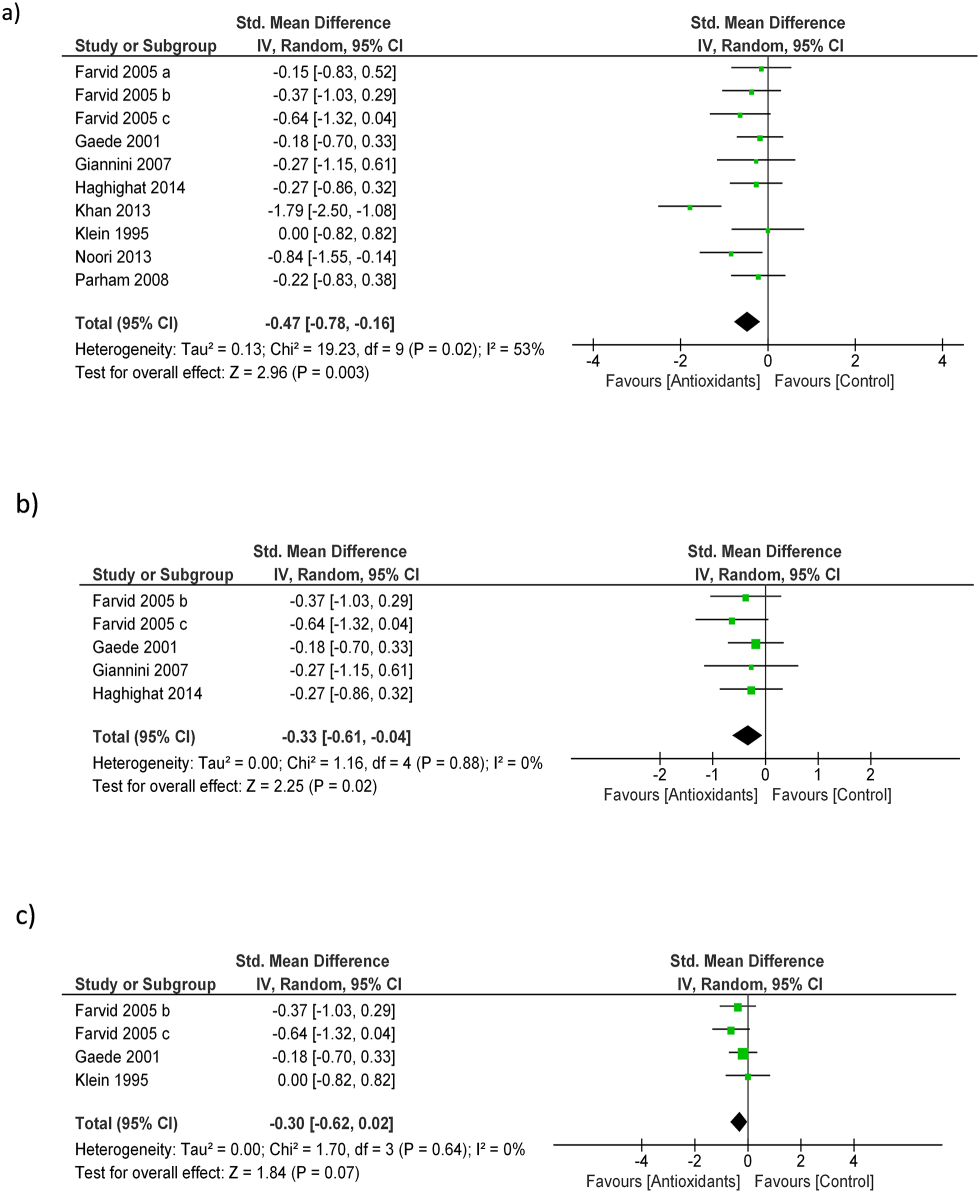
Effect of antioxidants vs. control on urinary albumin (2a); sensitivity analyses on separate effects by Vitamin E (2b) and Vitamin C supplements (2c).

**Fig 3:**
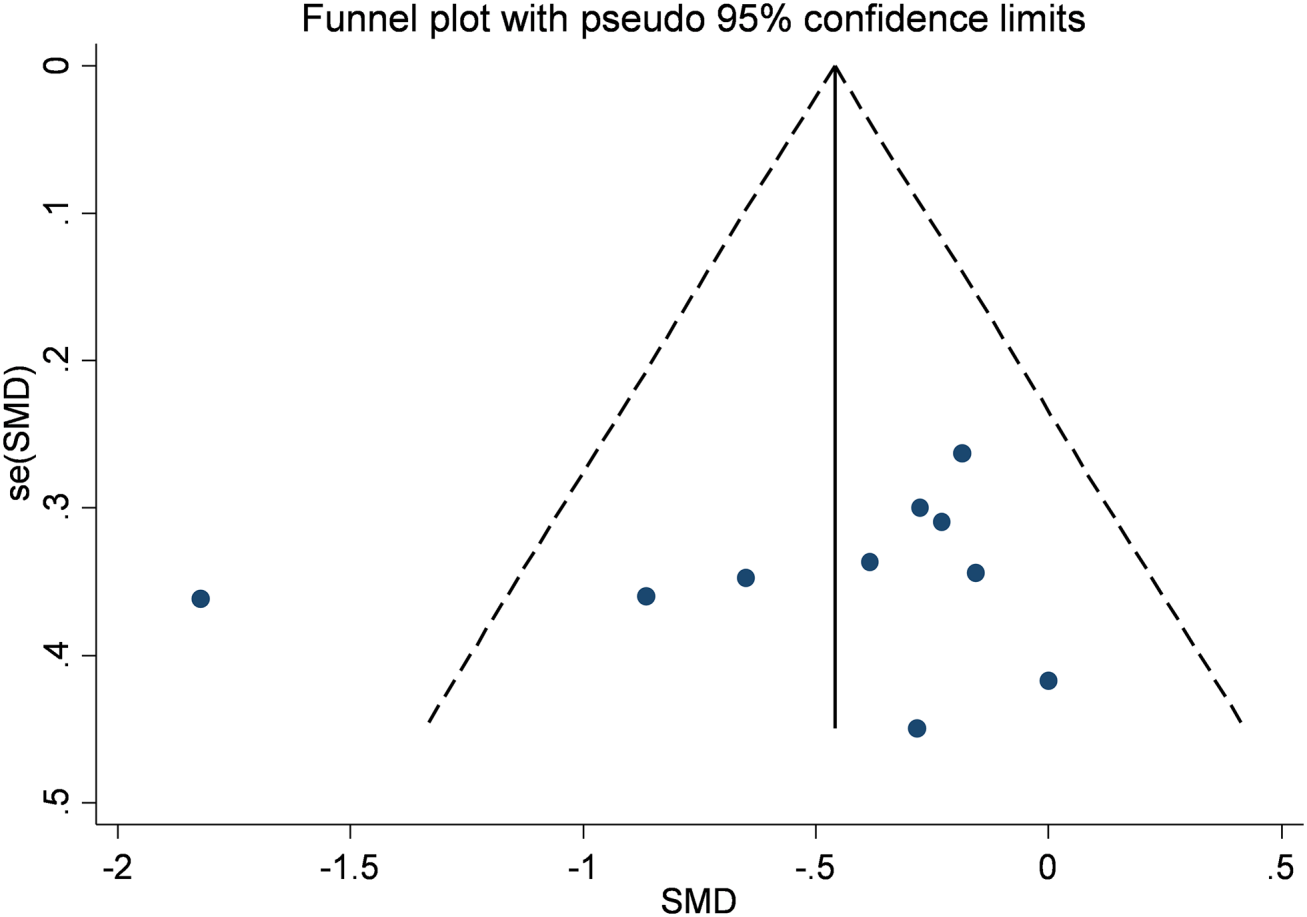
Publication bias (funnel plot) for urinary albumin.

In a pooled analysis of eight studies (327 patients) [13–20], antioxidant treatment produced a significant decrease in UAE levels as compared to control (SMD: -0.47; 95% CI -0.78, -0.16, Fig 2a). The analysis had mild level of heterogeneity (Chi^2^ = 19.23, df = 9 (P = 0.02); I^2^ = 53%) which was totally nullified in a sensitive analysis not including the only study with an open label design [18]. No publication bias was observed by visual inspection of the funnel plot and Egger’s regression test (Fig 3). The quality of the body of evidence for this outcome (GRADE) resulted high after being upgraded for strong magnitude of effects and downgraded for study limitations (very short follow-up of all the included studies) (Table 3).

**Table 3:** Summary of findings (GRADE) table.

Outcome	Effect estimate (95% CI)	N. of participants (studies)	Quality of the evidence (GRADE)
ESKD*	N/A	3654 (1 study)	N/A
UAE	SMD: -0.47 [-0.78, -0.16]	371 (8 studies)	⊕⊕⊕⊕^1^ High
Proteinuria*	N/A	76 (1 study)	N/A
Serum creatinine*	N/A	29 (1 study)	N/A
GFR (ml/min/1.73m^2^)	MD: -0.12 [-8.79, 8.54]	85 (3 studies)	⊕○○○^2^ Very Low

**GRADE Working Group grades of evidence: High quality**: Further research is very unlikely to change our confidence in the estimate of effect. **Moderate quality**: Further research is likely to have an important impact on our confidence in the estimate of effect and may change the estimate. **Low quality**: Further research is very likely to have an important impact on our confidence in the estimate of effect and is likely to change the estimate. **Very low quality**: We are very uncertain about the estimate. GFR: glomerular filtration rate; MD: mean difference; SMD: standardized mean difference; UAE: urinary albumin excretion

*: data from single studies and/or reported in a narrative way (outcome ungradable)

^1^: Upgraded for strong magnitude of effects; Downgraded for study limitation (very short follow-up)

^2^: Downgraded for study limitations (very short follow-up and small study populations) and evidence of imprecision (wide confidence intervals)

These findings were in line with results obtained by four single studies [21, 22, 25, 26], not suitable to be included in the meta-analysis. Conversely, two other studies [23, 24] did not report significant changes in UAE after antioxidant therapy as compared with placebo.

In subgroup analyses performed according to the type of antioxidant administered, benefits on UAE were confirmed in trials testing Vitamin E (4 studies/5 separate arms-194 participants; SMD—0.33 [-0.61, -0.04], Fig 2b) while the effect lost statistical significance in trials using vitamin C (3 studies/4 separate arms-152 participants; SMD -0.30 [-0.62, 0.02] Fig 2c).

Lonn et al [24] reported also information on the incidence of new cases of microalbuminuria which was statistically not different between the intervention- (35.3%) and the control group (37.5%) (p = 0.14).

#### Proteinuria

Farvid et al. [15] did not report significant changes in proteinuria after combined treatment with zinc + magnesium or Vitamin C + Vitamin E.

#### Serum creatinine

In one trial [14], no significant difference was observed in end of treatment serum creatinine values between the intervention and the control group (p = 0.55).

#### Renal function

**Fig 4:**
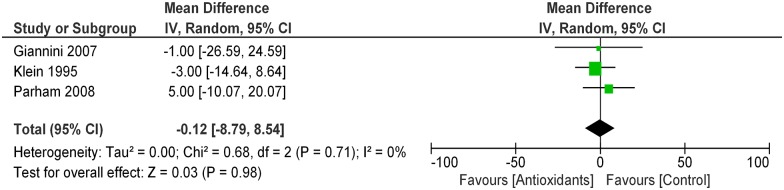
Effect of antioxidants vs. control on renal function (GFR).

In data pooled from 3 RCTs [13, 16, 17] (85 patients), antioxidant supplements had no tangible effects on GFR as compared with control (MD -0.12 ml/min/1.73m^2^; 95% CI -0.06, 0.01; Fig 4). Heterogeneity in this analysis was absent (Chi^2^ = 0.68, p = 0.71; I^2^ = 0%). The quality of the body of evidence (GRADE) for this outcome resulted very low after being downgraded for study limitations (very short follow-up and small study populations) and evidence of imprecision (wide confidence intervals) (Table 3). In one single study not suitable to be included in the meta-analysis [16], there was no significant difference in the end of treatment clearance of creatinine between individuals randomized to Vitamin E or placebo (p = 0.34).

#### Adverse events

Six RCTs did not report significant adverse events in both the active and control groups [13, 14, 16, 19, 20, 24]. In Parham et al. [17] two individuals apiece for each study group had episodes of epigastric pain. In Farvid et al. [15] two individuals in the active group withdrew because of side effects (not further specified) in the 1^st^ week of study. The remaining studies [18, 21–23, 25, 26], did not look systematically at side events.

## Discussion

This systematic review and meta-analysis aimed at assessing potential benefits of antioxidant agents on kidney disease progression in diabetic individuals with altered renal function. Unfortunately, despite diverse randomized controlled trials matching the review criteria were retrieved, the question as to whether antioxidant supplements should be systematically recommended for retarding DKD remains unclarified for various reasons.

First, scarce or no information was available on hard renal outcomes—that is kidney disease progression to end-stage as defined as the need for chronic dialysis or kidney transplantation. Conversely, the majority of studies were powered, both in terms of sample size and follow-up length, only to explore surrogate endpoints such as the absolute change in urinary albumin excretion or renal function. Second, only a minority of the included trials provided outcome data in a format suitable to be incorporated in meta-analyses while most information was confined to single studies or provided in a descriptive way only. This limitation hampered the robustness and generalizability of findings and prevented performing more complex, stratified statistical analyses according to various study characteristics, as originally pre-planned.

Third, despite heterogeneity in pooled analyses was unexpectedly low to moderate, there was high variability among trials with respect to population characteristics, study duration, severity of renal impairment and, above all, type and dose of antioxidant administered. No less important, most RCTs were of low methodological quality and risk of bias was unclear for the majority of the items analyzed, hence limiting the overall quality of evidence available.

In a meta-analysis of eight studies (371 individuals), antioxidants administration led to a significant reduction in albuminuria as compared to control. Such a finding was consistent with those obtained by four single RCTs [21, 22, 25, 26] not suitable to be included in the meta-analysis and echoed previous observations reported by a wealth of evidence based on uncontrolled or non-randomized studies [28–32]. Pathological urinary albumin excretion (UAE) is one of the most robust and earliest signs of diabetes-induced kidney injury. This abnormality is originally consequent to a damaged glomerular filtration barrier resulting in an increased permeability to plasma proteins. Interestingly, all pathological changes underlying UAE worsening, including abnormal renal hemodynamics, glomerular basement membrane thickening, mesangial expansion, extracellular matrix accumulation and glomerulosclerosis, are largely driven by diabetes-induced ROS, setting the rationale for treatments targeting enhanced oxidative stress [33].

Of note, in this meta-analysis the observed reduction in albuminuria was as high as -0.33 [SMD, 95% CI -0.61, -0.04], there was a quite strong magnitude of effects and there was no evidence of publication bias. Taken all together, these observations might be of high importance in terms of potential clinical impact of antioxidant treatments in DKD patients. Although enthusiastic, however, these findings must necessary be toned down in the wake of the (still) exploratory nature, the low number and the above-mentioned limits of the included trials, being rather more useful for setting the stage for confirmatory studies. New evidence appears also necessary for clarifying whether the observed benefits may be attributable to the whole class of antioxidants rather than to single compounds. For instance, with respect to UAE, this latter possibility would be suggested by sensitive analyses showing a persistence of benefits in trials testing vitamin E and an apparent lack of effects in those testing vitamin C.

As aforementioned, another important aim of this review was also to ascertain possible direct benefits of antioxidant agents on renal function. In a previous Cochrane review focusing on chronic kidney disease of various etiology, antioxidant therapy significantly reduced incidence of ESKD, serum creatinine levels and improved creatinine clearance, although findings relied on few trials of suboptimal quality and a very small number of events [34].

In the diabetic setting, preliminary evidence exists indicating that antioxidant supplementation may, in some cases, normalize GFR or creatinine clearance, particularly in individuals with hyperfiltration [35–37].

In our review, such benefits were apparently not confirmed in a pooled analysis of three studies (85 patients) and in two other single studies [14, 16] not reporting significant variations in GFR or serum creatinine after antioxidant therapy.

As persistent changes in renal function are notoriously achieved by long-term treatments, this lack of effect may be influenced by the very short therapy duration and the low number of patients with significantly impaired renal function at baseline, leaving the issue of direct benefits of antioxidant therapy on renal function still open.

Our review has strengths and limitations that deserve mentioning. Strengths are a pre-published protocol and a literature search, study selection, data extraction, cumulative analyses, bias and quality assessment that have all been performed following current best methodological standards for systematic reviews.

As alluded to before, the key limitation of this review mostly relies on the robustness of information available from the majority of the included studies. All the reviewed studies but one were single-center and long-term data on clinically relevant outcomes, such as need for dialysis or kidney transplantation and change in renal function, were basically lacking.

Alternative strategies including the adoption of measured GFR or other valid, non-surrogate estimates of renal function/damage might be a way for future trials for overcoming the need for large sample sizes and long follow-up times [38].

Furthermore, the short follow up period may prevent establishing durability of the observed effects (e.g. on UAE) and the quite high heterogeneity among study cohorts may limit the generalizability and applicability of findings to the whole DKD population.

In conclusion, despite cumulative findings point at some benefits of antioxidant therapy (particularly vitamin E) on early signs of renal damage, there is still no robust evidence supporting a widespread use of any of these agents as an alternative (or additive) therapy for retarding diabetic kidney disease progression. Future studies targeting hard rather than surrogate endpoints and with longer follow-up and larger sample size are advocated.

## Supporting information

S1 File PRISMA checklist of this systematic review. (DOC)

S1 Table Search strategy in CENTRAL, Ovid-Medline and PubMed databases. (DOCX)
