# Supplementary material for: Antioxidant agents for delaying diabetic kidney disease progression: A systematic review and meta-analysis
Source: PLoS One. 2017 Jun 1;12(6):e0178699. doi: 10.1371/journal.pone.0178699 (PMC5453586; doi:10.1371/journal.pone.0178699)
Supplement: S1 Table — (DOCX) [file pone.0178699.s002.docx]

**Supplementary Table S1**. Search strategy in CENTRAL, Ovid-Medline and PubMed databases (up to date as November 15, 2016)

| **CENTRAL** |
| --- |
| 1. MeSH descriptor Diabetes Mellitus, this term only 2. MeSH descriptor Diabetes Mellitus, Type 1 explode all trees 3. MeSH descriptor Diabetes Mellitus, Type 2 explode all trees 4. MeSH descriptor Diabetic Nephropathies explode all trees 5. diabet*:ti,ab,kw 6. (niddm or iddm):ab,ti,kw 7. (#1 or #2 or #3 or #4 or #5 or #6) 8. MeSH descriptor Antioxidants, this term only 9. MeSH descriptor Antioxidants, explode all trees 10. antioxidant*:ab,ti,kw 11. MeSH descriptor Vitamin A, explode all trees 12. MeSH descriptor Vitamin A, this term only 13. vitamin a:ab,ti,kw 14. carotenoid*:ti,ab,kw 15. MeSH descriptor carotenoids, explode all trees 16. MeSH descriptor carotenoids, this term only 17. MeSH descriptor vitamins, explode all trees 18. MeSH descriptor vitamins, this term only 19. beta-carotene:ti,ab,kw 20. retinol:ti,ab,kw 21. MeSH descriptor vitamin c, explode all trees 22. MeSH descriptor vitamin c, this term only 23. vitamin c:ab,ti,kw 24. ascorbic acid:ab,ti,kw 25. ascorbate:ab,ti,kw 26. MeSH descriptor vitamin e, explode all trees 27. MeSH descriptor vitamin e, this term only 28. vitamin e:ab,ti,kw 29. tocopherol:ab,ti,kw 30. selenium:ab,ti,kw 31. zinc:ab,ti,kw 32. methionine:ab,ti,kw 33. ubiquinone:ab,ti,kw 34. Coenzyme Q10:ab,ti,kw 35. ubidecarenone:ab,ti,kw 36. coenzyme Q:ab,ti,kw 37. CoQ10:ab,ti,kw 38. (#8 or #9 or #10 or #11 or #12 or #13 or #14 or #15 or #16 or #17 or #18 or #19 or #20 or #21 or #22 or #23 or #24 or #25 or #26 or #27 or #28 or #29 or #30 or #31 or #32 or #33 or #34 or #35 or #36 or #37) 39. #7 and #38 |
| **OVID-Medline** |
| 1. Diabetes Mellitus/ 2. exp Diabetes Mellitus, Type 1/ 3. exp Diabetes Mellitus, Type 2/ 4. Diabetic Nephropathies/ 5. diabet$.ti,ab,tw,kw 6. (niddm or iddm).ti,ab,tw,kw 7. exp Blood Glucose/ 8. exp Hyperglycemia/ 9. exp Hemoglobin A, Glycosylated/ 10. (blood glucos$ or hyperglyc?emi$ or h?emoglobin$ A).ab,ti,kw,tw 11. (HbA1C or Hb A or HbA 1c or HbA or A1Cs).ab,ti,tw,kw 12. (glycosylated adj6 h?emoglobin$).ab,ti,tw,kw 13. (glucos$ adj3 management$).ab,ti,tw,kw 14. OR (1-13) 15. exp antioxidants 16. Antioxidant* .ab,ti,tw,kw 17. beta-carotene .ab,ti,tw,kw 18. exp vitamin a/ 19. exp carotenoids/ 20. exp vitamins/ 21. vitamin A.ab,ti,tw,kw 22. retinol.ab,ti,tw,kw 23. exp vitamin c/ 24. vitamin C .ab,ti,tw,kw 25. ascorbic acid.ab,ti,tw,kw 26. ascorbate.ab,ti,tw,kw 27. exp vitamin e/ 28. vitamin E.ab,ti,tw,kw 29. tocopherol.ab,ti,tw,kw 30. selenium.ab,ti,tw,kw 31. zinc.ab,ti,tw,kw 32. methionine.ab,ti,tw,kw 33. ubiquinone.ab,ti,tw,kw 34. Coenzyme Q10.ab,ti,tw,kw 35. ubidecarenone.ab,ti,tw,kw 36. coenzyme Q.ab,ti,tw,kw 37. CoQ10.ab,ti,tw,kw 38. OR (15-37) 39. 14 AND 38 40. Limit 39 to Humans 41. (comment or editorial or historical-article).pt. 42. 40 not 41 43. randomized controlled trial.pt. 44. controlled clinical trial.pt. 45. randomi?ed.ab,ti,kw,tw 46. placebo$.ab,ti,tw,kw 47. drug therapy.fs. 48. randomly.ab,ti,tw 49. trial$.ab,ti.tw,kw 50. group$.ab,ti,tw 51. Cross-over Studies/ 52. (crossover or cross-over).tw. 53. OR (43-52) 54. 42 AND 53 |
| **PubMed** |
| (((diabetes mellitus OR diabetic nephropath*) AND (antioxidant* OR vitamin* OR vitamin A OR vitamin C OR vitamin E OR selenium OR zinc OR methionine OR ubiquinone) AND ((Clinical Study[ptyp] OR Clinical Trial[ptyp] OR Comparative Study[ptyp] OR Controlled Clinical Trial[ptyp] OR Multicenter Study[ptyp] OR Pragmatic Clinical Trial[ptyp] OR Randomized Controlled Trial[ptyp]) AND "humans"[MeSH Terms]))) |
